# Differential effects of the stress peptides PACAP and CRF on sleep architecture in mice

**DOI:** 10.1038/s44277-024-00003-y

**Published:** 2024-03-19

**Authors:** Allison R. Foilb, Elisa M. Taylor-Yeremeeva, Emma L. Fritsch, Caitlin Ravichandran, Kimberly R. Lezak, Galen Missig, Kenneth M. McCullough, William A. Carlezon

**Affiliations:** Basic Neuroscience Division, Department of Psychiatry, Harvard Medical School, McLean Hospital, Belmont, MA USA

**Keywords:** Stress and resilience, REM sleep

## Abstract

Stress produces profound effects on behavior, including persistent alterations in sleep patterns. Here we examined the effects of two prototypical stress peptides, pituitary adenylate cyclase-activating polypeptide (PACAP) and corticotropin-releasing factor (CRF), on sleep architecture and other translationally-relevant endpoints. Male and female mice were implanted with subcutaneous transmitters enabling continuous measurement of electroencephalography (EEG) and electromyography (EMG), as well as body temperature and locomotor activity, without tethering that restricts free movement, body posture, or head orientation during sleep. At baseline, females spent more time awake (AW) and less time in slow wave sleep (SWS) than males. Mice then received intracerebral infusions of PACAP or CRF at doses producing equivalent increases in anxiety-like behavior. The effects of PACAP on sleep architecture were similar in both sexes and resembled those reported in male mice after chronic stress exposure. Compared to vehicle infusions, PACAP infusions decreased time in AW, increased time in SWS, and increased rapid eye movement sleep (REM) time and bouts on the day following treatment. In addition, PACAP effects on REM time remained detectable a week after treatment. PACAP infusions also reduced body temperature and locomotor activity. Under the same experimental conditions, CRF infusions had minimal effects on sleep architecture in either sex, causing only transient increases in SWS during the dark phase, with no effects on temperature or activity. These findings suggest that PACAP and CRF have fundamentally different effects on sleep-related metrics and provide new insights into the mechanisms by which stress disrupts sleep.

## Lay summary

Exposure to stress and trauma causes short- and long-term disruptions in sleep. Understanding the brain chemicals that cause these effects might enable new treatment options. We studied in isolation the sleep-related effects of two stress peptides: PACAP (pituitary adenylate cyclase-activating polypeptide) and CRF (corticotropin-releasing factor). We found that PACAP caused long-lasting sleep disruptions resembling those caused by stress, whereas CRF effects were mild and transient, suggesting that PACAP plays a prominent role stress-induced sleep disruption.

## Introduction

Sleep disruption is a diagnostic criterion for stress-related conditions including major depressive disorder (MDD), generalized anxiety disorder (GAD), and post-traumatic stress disorder (PTSD) [1]. Dysfunctional sleep encompasses a range of problems including excessive sleep, diminished sleep, or disrupted and fragmented (comprising shorter bouts) sleep. Research in humans and laboratory animals provides extensive evidence that stress and sleep have a reciprocal relationship [2–8]. Stress often dysregulates sleep and circadian patterns [3–6, 9, 10], and conversely, abnormal sleep can serve as a form of stress, exacerbating symptom severity in individuals with stress-related conditions [2–5]. Sleep dysregulation in individuals with stress-related conditions also increases risk for substance use disorders, as many attempt to self-medicate sleep deficits [11]. An improved understanding of interactions between stress and sleep will help to advance the ability to diagnose, treat, and even prevent numerous forms of psychiatric illness.

Sleep as a metric has many characteristics, including the ability to measure the same endpoints across species, enhancing alignment of clinical and neuroscience research on mental health conditions [8, 12, 13]. Studies in rodents demonstrate that various forms of stress can produce alterations in sleep architecture. For example, footshock and immobilization stress alter diurnal patterns of rapid eye movement (REM) and non-REM (NREM) sleep [9]. Our lab has examined the effects of chronic social defeat stress (CSDS), an ethological form of stress that involves physical and emotional elements, on sleep in male mice and found alterations in all vigilance states measured: decreases in active wakefulness (AW), increases in slow wave sleep (SWS) and increases in REM [10, 14]. In addition, CSDS also disrupted the rhythmicity of body temperature and locomotor activity, such that the normal daily amplitude (rhythm strength) of these endpoints was reduced (flattened) [10]. Importantly, the changes in REM bouts and body temperature persisted beyond the termination of the stressor [10, 14, 15]. These long-lasting effects of stress are notable because they are commonly observed in individuals with MDD [4, 16–19], and their persistence suggests translational relevance in the context of modeling stress-induced psychiatric illnesses, which are by definition persistent and disruptive [1]. The increased use of translationally-relevant endpoints such as sleep and body temperature—among others—in rodents may improve the ability of model systems to more accurately predict outcomes in humans [8, 12, 13].

The mechanisms by which stress triggers psychiatric illness remain unclear [1, 8], impeding development of improved therapeutics. Pituitary adenylate cyclase-activating polypeptide (PACAP) and corticotropin-releasing factor (CRF) are peptides with well-validated roles in the biology of stress and stress-related conditions, including mood and anxiety disorders [13, 20–25]. Preclinical studies demonstrate that both peptide systems are activated and altered by stress, and administration of either peptide produces stress-like effects [13, 20, 24]. Both PACAP and CRF are highly conserved across species and produce similar stress-like behavioral effects, including increases in acoustic startle, a measure of vigilance commonly used assess anxiety and fear [21, 26–28]. Notably, while acute treatment with CRF produces enhancements in startle and fear responses that resolve within 24 h of treatment, those produced by acute PACAP treatment can persist for a week or more [21, 28–30]. Genetic differences in PACAP and CRF systems are also found in individuals vulnerable to stress [22–24, 31, 32]. Despite well-characterized sex differences in PACAP and CRF systems that may correspond with the prevalence of stress-related psychiatric disorders in clinical populations, few preclinical studies examining these peptides in parallel include both males and females [20, 22, 33–42]. Moreover, the contributions of stress peptides such as PACAP and CRF to stress-induced changes in sleep architecture are not well understood. There is evidence that both PACAP and CRF impact biological rhythms and sleep, although the findings are often inconsistent or conflicting across studies [9, 43–49]. In general, existing studies indicate opposite roles of PACAP and CRF on sleep endpoints such as REM. Some reports indicate that acute CRF treatment reduces REM, whereas PACAP increases REM [43, 45–47, 50]. The contrasting effects of PACAP and CRF on REM is surprising due to their similar behavioral effects and evidence that PACAP upregulates CRF expression and production [51–53]. Microinfusion of PACAP directly into the pons, a brain region involved in regulation of REM, produces alterations in sleep for a week after treatment, consistent with the persistent effects of PACAP on other behaviors [21, 30, 47, 54]. While stress can produce persistent and often intractable effects on sleep [8], the ways in which CRF might contribute to these effects have not been thoroughly characterized nor directly compared to those of PACAP [10].

The present studies were designed to characterize the contributions of PACAP and CRF in the regulation of sleep architecture, body temperature, and locomotor activity in male and female mice. First, we identified doses of each peptide that cause equivalent increases in anxiety-related behavior in the elevated plus maze (EPM), enabling physiologically-relevant comparisons in sleep studies. Then, in separate cohorts of mice, we used a wireless telemetry system and subcutaneous transmitters that enable continuous measurement of electroencephalography (EEG), electromyography (EMG), body temperature, and locomotor activity without tethering that might restrict movement, posture, or head orientation [10, 14, 55, 56] to examine the effects of each peptide on sleep architecture. Male and female mice were given intracerebral ventricular (ICV) infusion of PACAP, CRF, or vehicle (aCSF) and studied for one week after treatment. For each mouse, we used the continuous data sets to quantify vigilance states (AW, SWS and REM), body temperature, and locomotor activity prior to treatment and in 24-h periods immediately following treatment and one week later.

## Methods

### Subjects

Adult (6–8 weeks) male and female C57BL/6 mice (Jackson Laboratories, Bar Harbor, ME) were housed 3–5 per cage until surgery. Colony rooms were temperature-controlled and maintained on a 12-h light/dark cycle (lights-on at 07:00). Mice had ad libitum food and water. Procedures were approved by McLean Hospital Institutional Animal Care and Use Committee and performed in accordance with the National Institutes of Health’s (NIH) Guide for the Care and Use of Animals.

### Surgery

Mice were anesthetized with intraperitoneal (IP) injections of 100 mg/kg ketamine/10 mg/kg xylazine mixture in saline. Stainless steel guide cannula (26-gauge, P1 Technologies, Roanoke, VA) were implanted for intracerebroventricular (ICV) injection of peptides (relative to bregma: −0.2 mm anterior/posterior, +1.0 mm medial/lateral, −2.4 mm dorsal/ventral). Dummy stylets without projection were used to ensure patency. Transmitters (F20-EET; Data Sciences International [DSI], St. Paul, MN) were implanted to enable EEG and EMG collection as described [10, 14, 55]. EEG leads were attached to the skull over the frontal lobe (+1.00 mm anterior/posterior, +1.00 mm medial/lateral) and contralateral parietal lobe (−3.00 mm anterior/posterior, −3.00 mm medial/lateral) via screws contacting dura. Electrodes and cannula were secured in place using dental cement. Antibiotic ointment was applied to the sutured incision, antibiotic (sulfamethoxazole and trimethoprim) was provided in water, and ketoprofen analgesic (5.0 mg/kg) was administered subcutaneously. Mice were singly-housed after surgery and given two weeks for recovery before physiological recordings began.

### Peptide infusions

Mice were acclimated to handling a week prior to infusions, and stylets were removed to promote familiarization with the procedures. Infusions occurred between 09:00-10:00, during the third hour of lights-on. Mice were divided into 3 treatment conditions: Vehicle, PACAP, and CRF. Peptide dosages were shown previously to produce equivalent behavioral (anxiogenic-like) effects in the EPM (see below). PACAP-38 (0.25 μg; Bachem, Torrance, CA) and CRF (1.0 μg; Bachem, Torrance, CA) were dissolved in artificial cerebrospinal fluid (aCSF; Harvard Apparatus, Holliston, MA) and administered via an internal cannula projecting 1.0 mm beyond the guide cannula at a volume of 1.0 μL and a rate of 0.5 μL/minute, with an additional 2 min to enable diffusion. Vehicle-treated mice received infusion of aCSF at the same volume and rate.

### Physiological recordings

Transmitter-implanted mice were housed in standard plastic cages that sat upon receiver platforms (RPC-1; DSI) for wireless data collection, as described [10, 14, 55]. Continuous collection of EEG, EMG, locomotor activity and body temperature occurred for four days prior to treatment, and one week after treatment. Quantification of vigilance stages was determined by a trained scorer blind to treatment condition using Neuroscore (DSI).

### EPM testing

To enable physiologically-relevant comparisons between PACAP and CRF in the sleep studies, we first used an independent cohort of male and female mice to identify dosages of each peptide that produce equivalent behavioral (anxiogenic-like) effects in the EPM [57]. Vehicle, PACAP, or CRF was administered via ICV cannula as described, between 09:00-10:00, 30 min before testing. Tests were performed using a standard apparatus (30-cm arms, 5.5 ×5.5-cm center area, 5.5-cm walls, elevated 80 cm); mice were placed in the center to start 5-minute tests. Sessions were videotaped and behavior (e.g., time spent on open arms) was quantified using EthoVision (https://www.noldus.com/ethovision-xt), as described [14].

### Statistical analyses

Data were analyzed using Graphpad Prism 9 with significance set to *P* < 0.05. Outliers were identified with ROUT outlier detection test (Q = 1); exclusions are noted below (Results). As recommended, sexes were combined when there were no sex differences [58]. EPM tests were analyzed using one-way ANOVAs. Baseline vigilance states were quantified for the total 24-h period prior to treatment (“Baseline”), and analyses were performed on data expressed as %Baseline during the 24 h immediately after treatment (10:00AM-10:00AM; “Day 1”) and during the same 24-h window 1 week after treatment (“Day 7”). Since the infusions were performed in the morning, the initial potion of the light phase included both pre- and post-infusion data; to enable comparisons between equivalent time periods for analyses that separate data into light and dark phases, light cycle-specific changes were calculated using the 9 h of lights-on after infusion (“Light”) and the subsequent 9 h of lights-off (“Dark”), thereby comprising the 18 consecutive hours after treatment. Light/Dark times at Baseline and Day 7 corresponded with Day 1. Changes in vigilance state duration and bouts, body temperature and locomotor activity were compared via mixed effects analyses to account for missing data at Day 7 due to transmitter malfunction in three mice. T-tests were used for within condition comparisons to Baseline, and within each vigilance state for EEG power analyses. One-way ANOVAs were used to compare changes in EEG absolute power across treatment conditions. Amplitude of diurnal fluctuations in body temperature and activity were calculated by fitting a cosinor model for timepoint, using R software (versions 4.2.3) and the *cosinor* package [59]. Representative EEG spectrograms were created using a freely-available multitaper spectral analysis code in Python 3.7.9 [60], with appropriate adjustments (i.e. sampling rate 500 Hz).

**Fig. 1:**
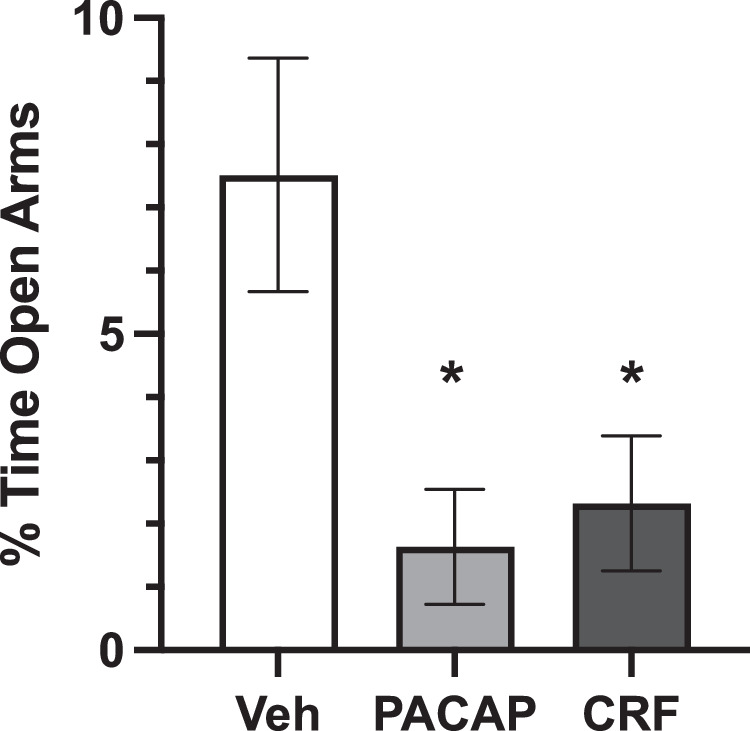
Stress peptide effects on EPM. Percentage of time (±SEM) spent in open arms during 5 min EPM after treatment with Vehicle (0.0, white), PACAP (0.25 µg, gray) or CRF (1.0 µg, black) in males and females combined. *P < 0.05 compared to Vehicles.

Significant effects were further analyzed with post-hoc comparisons: Tukey’s post-hoc tests were used to compare across all conditions and timepoints; where noted, Dunnett’s post-hoc tests were used to make comparisons to Vehicle or Baseline. The EEG data channel was lost in three Vehicle-treated male mice before Day 7, preventing vigilance state assessment at that timepoint; prior timepoints, as well as all temperature and activity data, are included for these mice.

## Results

### Effects of PACAP and CRF in the EPM

The EPM experiment was designed to identify dosages of PACAP and CRF that produce equivalent anxiogenic-like effects [57], enabling physiologically-relevant comparisons in subsequent sleep studies. Dosages were selected based on pilot studies of these peptides in other procedures (Unpublished data, Carlezon lab). Mice were placed in the center of the EPM 30 min after ICV injection of Vehicle, PACAP (0.25 µg), or CRF (1.0 µg). ROUT with Q = 1 was used to identify outliers, and led to the exclusion of 1 Vehicle male, 2 PACAP males, 1 PACAP female, 1 CRF male, and 1 CRF female; the final numbers of subjects were Vehicle (6 males, 6 females), PACAP (5 males, 6 females), and CRF (7 males, 5 females). Analyses revealed no sex differences—there was no main effect of Sex (F(1,29) = 0.44, *P* = 0.51, not significant [n.s.]) and no Sex x Condition interaction (F(2,29) = 1.11, *P* = 0.34, n.s.) (not shown)—justifying combining the data across sexes to increase power. With sexes combined, there was a main effect of Condition (F(2,29) = 5.85, *P* = 0.007), with both PACAP and CRF reducing %time in open arms, relative to vehicle-treated mice (*P* = 0.012 and 0.024, respectively; Tukey’s tests) (Fig. 1). There were no differences between the PACAP and CRF conditions (*P* = 0.95, n.s.)—indeed, the group means were virtually equivalent—providing a justification for selecting these peptide dosages for the sleep studies.

**Fig. 2:**
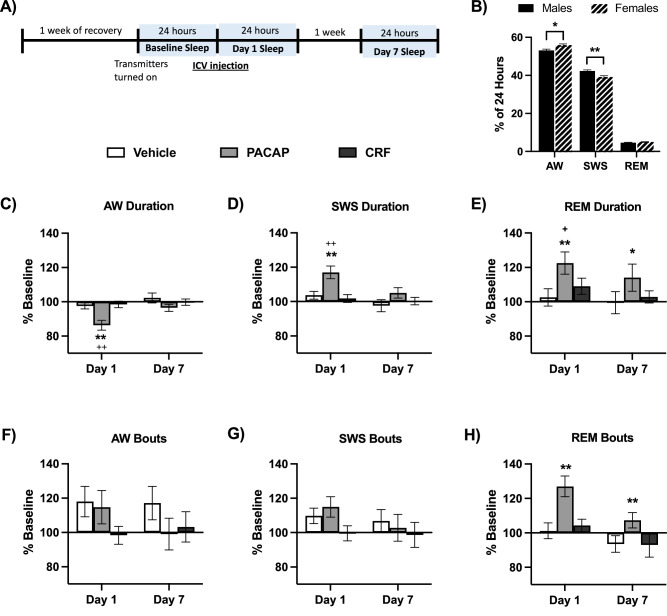
Alterations in vigilance states. **A** Timeline of experiment and timepoints of vigilance state assessment. **B** Average (+SEM) duration of vigilance states at Baseline, prior to peptide treatment with males (solid black bars) and females (striped bars) significantly differing at Baseline in duration of wake (AW) and slow wave sleep (SWS), but not rapid eye movement sleep (REM). Display changes as percentage of Baseline (±SEM) after Vehicle (white), PACAP (gray) and CRF (black) treatment of **C** AW duration, **D** SWS duration, **E** REM duration, **F** AW bouts, **G** SWS bouts, and **H** REM bouts. Asterisks indicates significant differences from Baseline vigilance states, plus signs indicate significant differences compared to Vehicle-treated mice. *^+^*P* < 0.05, ** ^++^*P* < 0.01.

### Baseline sex differences in vigilance states

The overall experimental design is depicted in Fig. 2A. Baseline vigilance states were assessed in the 24 h prior to treatment. Unpaired, two-tailed t-tests were used to compare durations of each vigilance state in males (*n* = 27) to those of females (*n* = 25). Analyses indicated significant sex differences in duration of AW (t(50) = 2.86, *P* = 0.006), and SWS (t(50) = 3.22, *P* = 0.0009), with increased AW and decreased SWS in females. There were no sex differences in duration of REM (t(50) = 1.07, *P* = 0.29, n.s.) (Fig. 2B).

**Fig. 3:**
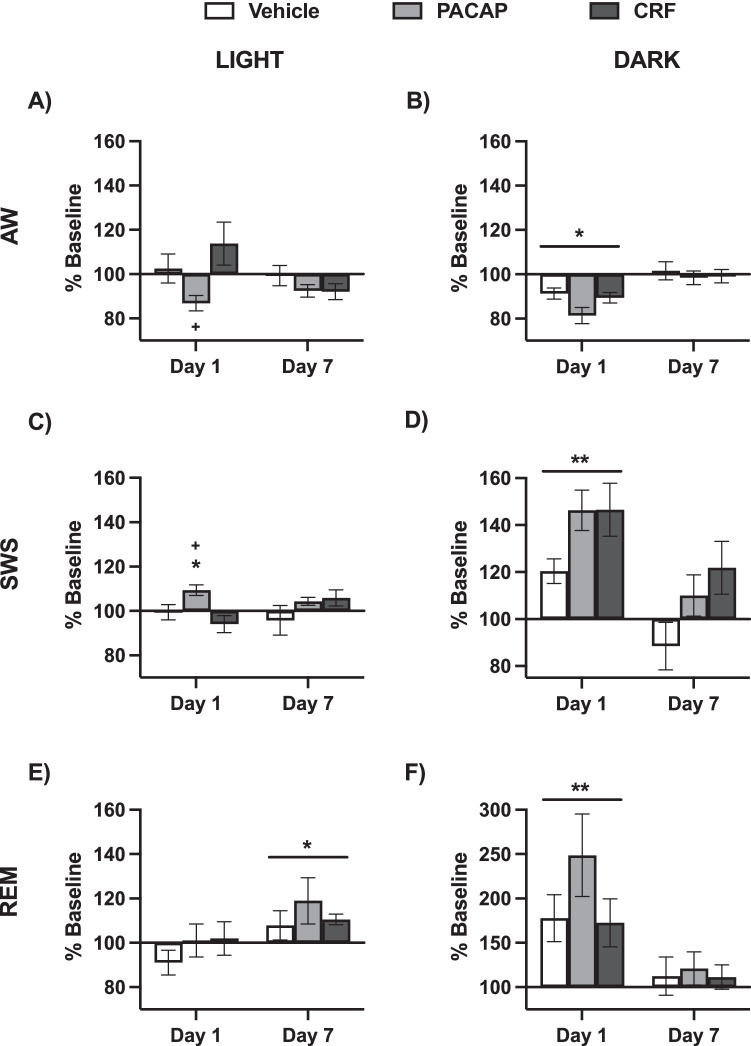
Light/Dark phase changes in vigilance states. Changes as percentage of Baseline (±SEM) after Vehicle (white), PACAP (gray) and CRF (black) treatment in duration of **A** AW during the Light phase, **B** AW during the Dark phase, **C** SWS during the Light phase, **D** SWS during the Dark phase, **E** REM during the Light phase, and **F** REM during the Dark phase. Asterisks indicates significant differences from Baseline vigilance state, asterisks over horizontal bars indicates that timepoint significantly differs from all other timepoints, and plus signs indicate significant differences compared to Vehicle-treated mice. *^+^*P* < 0.05, **P < 0.01.

### Changes in vigilance states by peptide treatment

Effects of peptide treatment on vigilance states is calculated as %Baseline, as described previously [10]. Despite pre-existing sex differences in vigilance state durations, mixed effects analyses of Sex x Timepoint within Condition revealed no sex differences in changes from Baseline after PACAP or CRF treatment. There was a significant main effect of sex on AW and SWS of Vehicle-treated mice; see Table 1 for all within-Condition Sex x Timepoint comparisons of vigilance state durations. To increase power, males and females were combined in all other analyses to increase the total number of mice per Condition to Vehicle (*n* = 17; 9 males,/8 females), PACAP (*n* = 19; 10 males/9 females), and CRF (*n* = 16; 8 males/8 females).

**Table 1:** Mixed effects analyses of Timepoint by Sex within condition and vigilance state.

Condition	Vigilance state	Main effect of timepoint	Main effect of sex	Timepoint × Sex interaction
Vehicle	AW	F (2, 27) = 1.31, *P* = 0.29	F (1, 15) = 6.55, *P = **0.022**	F (2, 27) = 1.75, *P* = 0.19
	SWS	F (2, 27) = 1.51, *P* = 0.24	F (1, 15) = 5.76, *P = **0.03**	F (2, 27) = 1.51, *P* = 0.24
	REM	F (2, 27) = 0.18, *P* = 0.83	F (1, 15) = 1.02, *P* = 0.33	F (2, 27) = 0.83, *P* = 0.45
PACAP	AW	F (2, 34) = 12.64, **P < **0.0001**	F (1, 17) = 0.86, *P* = 0.37	F (2, 34) = 0.43, *P* = 0.65
	SWS	F (2, 34) = 10.17, **P = **0.0003**	F (1, 17) = 0.64, *P* = 0.43	F (2, 34) = 0.30, *P* = 0.74
	REM	F (2, 34) = 4.62, *P = **0.017**	F (1, 17) = 0.02, *P* = 0.90	F (2, 34) = 0.46, *P* = 0.64
CRF	AW	F (2, 28) = 0.34, *P* = 0.71	F (1, 14) = 0.84, *P* = 0.37	F (2, 28) = 0.61, *P* = 0.55
	SWS	F (2, 28) = 0.34, *P* = 0.71	F (1, 14) = 0.54, *P* = 0.47	F (2, 28) = 0.72, *P* = 0.50
	REM	F (2, 28) = 2.29, *P* = 0.12	F (1, 14) = 3.00, *P* = 0.11	F (2, 28) = 1.96, *P* = 0.16

A main effect of sex is found within the Vehicle condition in AW and SWS duration, and a main effect of Timepoint is observed in all vigilance states for PACAP mice.

*P < 0.05; **P < 0.001.

Using the combined data, we analyzed durations and number of bouts of each vigilance state by treatment Condition to assess the impact of the stress peptides on sleep. Changes in duration of sleep reflect excessive or diminished sleep and increases in SWS or REM bouts represent disrupted or fragmented sleep, as often observed in individuals with stress-related disorders [16, 59, 60]. A mixed effects analysis of Condition x Timepoint for AW duration revealed main effects of Timepoint (F(2,95) = 10.52, *P* < 0.0001), Condition (F(2,49) = 7.45, *P* = 0.0015), and a Timepoint x Condition interaction (F(4, 95) = 4.31, *P* = 0.003) (Fig. 2C). Post-hoc tests (Tukey’s) indicated that the Day 1 timepoint differed from Baseline and Day 7 (*P* = 0.0002 and 0.001, respectively). PACAP-treated mice differed from both Vehicle- and CRF-treated mice overall (*P* = 0.0034 and 0.008, respectively) and specifically at Day 1, corresponding with the 24-hour period after treatment (*P*’s < 0.0001). Within conditions, there were no differences between timepoints in Vehicle or CRF-treated mice, but PACAP-treated mice had reductions in AW duration at Day 1 compared to Baseline and Day 7 (*P*’s < 0.0001). A mixed effects analysis of AW bouts revealed no main effect of Timepoint (F(2,95) = 2.13, *P* = 0.125, n.s.), Condition (F(2,49) = 1.28, *P* = 0.288, n.s.), nor a Timepoint x Condition interaction (F(4,95) = 1.35, *P* = 0.258, n.s) (Fig. 2F). Reductions in duration without changes in the number of bouts indicates that the average length of bouts is shorter, reflecting fragmentation.

A mixed effects analysis of SWS duration also revelaed main effects of Timepoint (F(2,95) = 9.69, *P* = 0.0001), Condition (F(2,49) = 7.40, *P* = 0.0016), and a Timepoint x Condition interaction (F(4,95) = 3.74, *P* = 0.0071) (Fig. 2D). Post-hoc tests (Tukey’s) revealed that SWS at Day 1 differed from Baseline and Day 7 (*P* = 0.0003 and 0.002, respectively). PACAP-treated mice differed from both Vehicle- and CRF-treated mice overall (*P* = 0.0046 and 0.0062, respectively) and specifically at Day 1 (*P* = 0.0002 and <0.0001). Tukey’s tests for effects within conditions revealed no differences between timepoints in Vehicle or CRF-treated mice, but PACAP-treated mice had a different SWS duration at Day 1 compared to their Baseline (*P* < 0.0001) and Day 7 (*P* = 0.005). A mixed-effects analysis of SWS bouts revealed no effect of Timepoint (F(2,95) = 2.51, *P* = 0.087, n.s.), Condition (F(2,49) = 1.07, *P* = 0.35, n.s.), nor a Timepoint x Condition interaction (F(4,95) = 0.92, *P* = 0.46, n.s.) (Fig. 2G). The combination of longer SWS bouts and increased SWS duration suggests lack of fragmentation.

Analysis of REM duration revealed a main effect of Timepoint (F (2,95) = 5.49, *P* = 0.006), but no effect of Condition (F(2,49) = 3.04, *P* = 0.057, n.s.), nor a Timepoint x Condition interaction (F(4,95) = 1.68, *P* = 0.16, n.s.) (Fig. 2E). Within Conditions there were no changes from Baseline in Vehicle- or CRF-treated mice, but PACAP-treated mice had different REM durations at Day 1 (*P* = 0.004) and Day 7 (*P* = 0.039) compared to Baseline. Mixed effects analysis of REM bouts revealed main effects of Timepoint (F(2,95) = 10.56, *P* < 0.0001) and Condition (F(2,49) = 6.19, *P* = 0.004), and a Timepoint x Condition interaction (F(4,95) = 4.00, *P* = 0.005) (Fig. 2H). Post hoc comparisons (Tukey’s) revealed that Day 1 differed from Baseline and Day 7 (*P* = 0.0012 and 0.0002, respectively). PACAP-treated mice differed from both Vehicle and CRF conditions (*P* = 0.009 and 0.015), specifically at Day 1 (*P*’s < 0.0001 and *P* = 0.0006), as well as compared to CRF at the Day 7 timepoint (*P* = 0.046). Within conditions, REM bouts did not change across timepoints for Vehicle or CRF-treated mice while PACAP-treated mice had significantly increased bouts of REM compared to Baseline at both Day 1 (*P* < 0.0001) and Day 7 (*P* = 0.0003). Unlike the case with AW, however, the change (increase) in REM bouts corresponds with the increase in REM duration. This suggests consistency in the length of REM bouts (i.e., lack of fragmentation), and that PACAP-treated mice entered REM more frequently at Day 1 and Day 7 compared to Baseline.

### Vigilance state durations during the light cycle

In all mice, the ICV infusions were performed during the third hour of lights-on in a 12-h light/dark cycle. To better understand when the treatments produced effects on vigilance, we examined behavior during the light and dark phase of the diurnal cycle.

Changes during the light phase occurred in the 9 h immediately after treatment, while the dark phase includes the subsequent period of equivalent length (9 h) during the dark phase, representing the 18 consecutive hrs following infusion. Mixed effects analysis of AW duration in the light phase revealed a Timepoint × Condition interaction (F(4,95) = 3.77, *P* = 0.0069), but no main effects of Timepoint (F(2,95) = 2.04, *P* = 0.14, n.s.) nor Condition (F(2,49) = 2.99, *P* = 0.059, n.s.) (Fig. 3A). Post hoc comparisons (Tukey’s) revealed that PACAP-treated mice differed from Vehicle and CRF-treated mice on Day 1 (*P*’s = 0.030 and *P* < 0.0001, respectively), and that CRF-treated mice displayed significantly increased AW durations on Day 1 when compared to Day 7 (*P* = 0.017). During the dark phase of the light cycle, there was a main effect of Timepoint for AW duration (F (2,95) = 24.32, *P* < 0.0001), with Day 1 decreased compared to Baseline and Day 7 (*P*’s < 0.0001) (Fig. 3B).

Analysis of SWS duration in the light phase also revealed a Timepoint x Condition interaction (F(4,95) = 3.69, *P* = 0.0077), but no main effects of Timepoint (F(2,95) = 0.39, *P* = 0.68, n.s.) nor Condition (F(2,49) = 3.16, *P* = 0.051, n.s.) (Fig. 3C). Post hoc comparisons (Tukey’s) revealed that PACAP-treated mice differed from Vehicle and CRF-treated mice at Day 1 (*P* = 0.039 and 0.0008, respectively). Within conditions, there were no differences between timepoints in Vehicle mice, but PACAP-treated mice had increased SWS during the light phase at Day 1 compared to Baseline (*P* = 0.037) and CRF-treated mice had increased SWS at Day 7 compared to Day 1 (*P* = 0.013). During the dark phase of the light cycle, there were main effects of Timepoint, (F(2,95) = 24.86, *P* < 0.0001) and Condition (F(2,49) = 4.34, *P* = 0.018) for SWS duration, but no Timepoint × Condition interaction (F(4,95) = 1.77, *P* = 0.14, n.s.) (Fig. 3D). Dark-phase SWS differed at Day 1 compared to both Baseline and Day 7 (*P*’s < 0.0001). Between conditions, CRF-treated mice differed from Vehicle-treated mice (*P* = 0.021).

Analysis of REM duration in the light phase revealed a main effect of Timepoint (F(2,95) = 5.50, *P* = 0.0055) but not Condition (F(2,49) = 0.89, *P* = 0.42, n.s.), nor a Timepoint × Condition interaction (F(2,95) = 0.51, *P* = 0.73, n.s.) (Fig. 3E). Light phase REM duration on Day 7 timepoint differed from Baseline and Day 1 (*P* = 0.025 and 0.0077, respectively). During the dark phase, there was again a main effect of Timepoint (F(2,95) = 19.11, *P* < 0.0001), but not Condition (F(2,49) = 1.05, *P* = 0.36, n.s.). nor a Timepoint x Condition interaction (F(4,95) = 1.27, *P* = 0.29) (Fig. 3F). Dark phase REM duration at Day 1 differed from Baseline and Day 7 (*P*’s < 0.0001).

### Body temperature and locomotor activity

Changes in body temperature and activity often correspond with changes in vigilance states. Transmitter problems led to the loss of temperature data from one PACAP female at all timepoints and one Vehicle male at the Day 7 timepoint. A mixed effects analysis of average temperature found no main effects of Timepoint (F(2,95) = 0.76, *P* = 0.47, n.s.), Condition (F(2,49) = 1.741, *P* = 0.19, n.s.), nor a Timepoint x Condition interaction (F(4,95) = 1.12, *P* = 0.35, n.s.). Within-condition t-tests found no significant changes in Vehicle or CRF-treated mice, but PACAP-treated mice had reduced temperature on Day 1 compared to Baseline (t(17) = 2.98, *P* = 0.009).

**Fig. 4:**
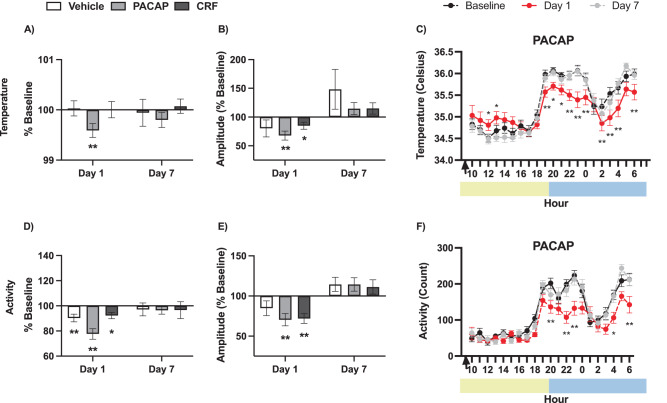
Changes in temperature and activity. **A** Average (±SEM) core body temperature at Day 1 and Day 7 as a percentage of Baseline. **B** Temperature amplitude (±SEM) at Day 1 and Day 7 as a percentage of Baseline. **C** Hourly core body temperature (±SEM) in degrees Celsius of PACAP-treated mice at Baseline (black), Day 1 (red), and Day 7 (gray). **D** Average (±SEM) activity at Day 1 and Day 7 as a percentage of Baseline. **E** Activity amplitude (± SEM) at Day 1 and Day 7 as a percentage of Baseline. **F** Hourly activity count (±SEM) of PACAP-treated mice at Baseline, Day 1, and Day 7. Asterisks indicates significant differences from Baseline, asterisks over horizontal bars indicate a main effect of Timepoint, plus signs indicate significant differences compared to Vehicle-treated mice. *^+^*P* < 0.05, **P < 0.01.

Analysis of body temperature in 1-h bins revealed characteristic decreases in body temperature during the light phase and increases in temperature at the start of the dark phase, with a dip in temperature midway through the dark phase [10, 55, 56]. In PACAP-treated mice, reductions in body temperature occurred during the dark phase of the light cycle, corresponding with treatment-induced increases in sleep when the mice are typically awake and active (Fig. 4B).

The amplitude of diurnal fluctuations in body temperature was analyzed with a cosinor model fit to hourly temperature data for each mouse and timepoint. Changes in rhythm were assessed as percentage of Baseline at Day 1 and Day 7, as previously [10]. Mixed-effects analysis of temperature amplitude found a main effect of Timepoint (F(2,95) = 10.63, *P* < 0.0001), but no main effect of Condition (F(2,48) = 0.89, *P* = 0.44, n.s.), nor a Timepoint ×Condition interaction (F(4,95) = 0.80, *P* = 0.53, n.s.). Post hoc comparisons (Tukey’s) revealed increased temperature amplitude on Day 7 compared to both Baseline (*P* = 0.04) and Day 1 (*P* < 0.0001). Within condition t-tests found reduced temperature amplitude on Day 1 compared to Baseline in PACAP-treated (t(17) = 4.13, *P* = 0.0007) and CRF-treated (t(15) = 2.46, *P* = 0.027) mice.

The patterns of diurnal fluctuations in locomotor activity resembled those observed in body temperature. Mixed-effects analysis found a main effect of Timepoint (F(2,98) = 12.98, *P* < 0.0001), but no main effect of Condition (F(2,49) = 1.56, *P* = 0.22, n.s.) nor a Timepoint x Condition interaction (F(4,98) = 1.94, *P* = 0.11, n.s.). T-test comparisons revealed reductions in locomotor activity levels in all conditions at Day 1 compared to Baseline: Vehicle (t(16) = 3.18, *P* = 0.006), PACAP (t(18) = 5.33, *P* < 0.0001), and CRF (t(15) = 3.07, *P* = 0.0077). Analysis of locomotor activity in 1-hr bins found reductions during the dark phase of the light cycle in PACAP-treated mice (Fig. 4D).

Activity amplitude was used to quantify changes in activity rhythms as a percentage of Baseline. A mixed effects analysis found a main effect of Timepoint (F(1,98) = 27.51, *P* < 0.0001), but not a main effect of Condition (F(2,49) = 0.44, *P* = 0.65, n.s.) nor a significant Timepoint x Condition interaction (F(4,98) = 0.50, *P* = 0.73, n.s.). Post hoc (Tukey’s) comparisons revealed differences in activity amplitude between each timepoint: Baseline compared to Day 1 (*P* < 0.0001) and Day7 (*P* = 0.29), as well as Day 1 compared to Day 7 (*P* < 0.0001). In addition, reductions in activity amplitude on Day 1 compared to Baseline were observed in PACAP- (t(17) = 3.68, *P* = 0.0018) and CRF-treated (t(15) = 5.24, *P* < 0.0001) mice.

### EEG power

EEG can also provide insight into patterns of neural activity, which change in response to stress, sleep drive, and sleep quality as well as cognitive demand [10, 61, 62]. EEG signals are also dysregulated in individuals with depression [62]. We focused on frequency bands corresponding to delta (0.5–4 Hz), theta (4–8 Hz), alpha (8–12 Hz), beta (16–24 Hz), and gamma (30–80 Hz) power. Changes in absolute EEG spectral power were calculated as a %Baseline at Day 1 for each powerband during each vigilance state.

**Fig. 5:**
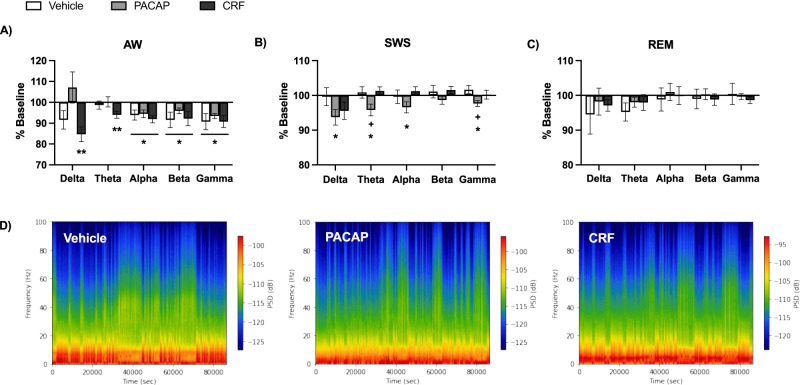
Changes in EEG absolute power by vigilance state. Average changes in EEG spectral power, displayed from low frequency (delta, 0.5–4 Hz) to high frequency (gamma, 30–80 Hz), at Day 1 as a percent of Baseline (±SEM) for **A** AW, **B** SWS, and **C** REM. **D** Representative spectrograms of EEG power at Day 1 from each treatment condition. Colors signify power spectral density (PSD) with cool to warm colors aligning with low to high power. Asterisks indicates significant differences from Baseline, plus signs indicate significant differences compared to Vehicle-treated mice. * ^+^*P* < 0.05, **P < 0.01.

For AW, one-way ANOVAs revealed treatment effects on delta power (F(2,49) = 4.20, *P* = 0.021) (Fig. 5A). Dunnett’s post-hoc comparisons of AW delta for PACAP- and CRF-treated mice to Vehicle mice found no differences. T-tests comparing Day 1 to Baseline within Condition and vigilance state revealed that Day 1 AW in CRF-treated mice differed from Baseline for delta (t(15) = 4.28, *P* = 0.0007) and theta (t(15) = 3.55, *P* = 0.0029) power. Alpha power at Day 1 differed from Baseline in all conditions: Vehicle, (t(16) = 2.57, *P* = 0.021), PACAP (t(18) = 2.91, *P* = 0.0094), and CRF (t(15) = 4.63, *P* = 0.0003). AW beta power also differed from Baseline at Day 1 in Vehicle (t(16) = 2.25, *P* = 0.039), PACAP (t(18) = 3.03, *P* = 0.0072), and CRF (t(15) = 2.36, *P* = 0.032), as did gamma: Vehicle (t(16) = 2.40, *P* = 0.029), PACAP (t(18) = 5.32, *P* < 0.0001), and CRF (t(15) = 3.26, *P* = 0.0053). These changes may reflect non-specific effects of the ICV injection during the light phase, which stimulates the mice when they are normally more likely to be sleeping. CRF-specific variations may further indicate disruption of diurnal rhythms in response to peptide treatment.

For SWS, one-way ANOVAs of each powerband revealed treatment effects on delta (F(2,49) = 4.07, *P* = 0.023) and gamma power (F(2,49) = 3.57, *P* = 0.035) (Fig. 5B). Dunnett’s post-hoc tests comparing PACAP- and CRF-treated mice to controls at these powerbands found that those given PACAP differed from Vehicle for both theta (*P* = 0.041) and gamma power (*P* = 0.021). Within condition t-tests found that PACAP-treated mice differed from Baseline at delta (t(18) = 2.80, *P* = 0.012), theta (t(18) = 2.50, *P* = 0.022), alpha (t(18) = 2.16, *P* = 0.044), and gamma powerbands (t(18) = 2.67, *P* = 0.016). Vehicle- and CRF-treated mice did not display changes from Baseline at any powerbands for SWS.

For REM, one-way ANOVAs and within conditions t-tests of EEG absolute power did not reveal any changes from Baseline at Day 1 for any condition (Fig. 5C). Spectrograms depicting all powerbands together across time (Day 1) for representative mice from each treatment condition are shown (Fig. 5D).

## Discussion

The present studies provide direct comparison of PACAP and CRF effects on sleep architecture under identical testing conditions, at dosages that cause comparable anxiogenic-like effects in mice. PACAP broadly impacted rhythms of sleep, body temperature, and locomotor activity. CRF, conversely, did not lead to major alterations in vigilance states, although there were small transient changes (e.g., SWS within the dark phase, EEG power during AW, and activity and temperature amplitude) that indicate efficacy of the dosage tested. The changes observed in PACAP-treated mice closely resemble those previously observed in CSDS-exposed mice: decreased AW, increased SWS, increased REM sleep, and reduced amplitude of body temperature and locomotor activity rhythms [10]. Some changes were seen across all conditions, including increased locomotor activity in the 24 h after injection, decreased wake/increased sleep during the dark phase of the light cycle and reduced alpha, beta and gamma power during AW. These changes might be non-specific, due to disrupted sleep early in the light phase of the diurnal light cycle (after ICV injections were administered) or the mild stress of ICV infusions (despite prior handling). Changes in vigilance state durations occurred primarily in the dark phase of the light cycle, with increased sleep and decreased wake when mice are typically awake and active. Within PACAP-treated mice, changes were also observed in the light phase of the light cycle, with the same pattern of decreased wakefulness and increased sleep. Corresponding with decreased time awake, PACAP-treated mice displayed an overall decrease in body temperature and locomotor activity during the 24 h after treatment, specifically during the dark phase of the light cycle, over 9 h after PACAP administration. Decreased temperature and activity amplitude at Day 1 (24 h after infusion) indicates PACAP and CRF each flatten normal diurnal body temperature and activity rhythms. This finding is consistent with previous work characterizing the effects of CSDS on these endpoints in mice [10], as well as clinical studies of individuals diagnosed with depression [17–19].

Changes in EEG spectral power during SWS were found in PACAP-treated mice at nearly all frequencies, and in CRF-treated mice during Wake. Reduction and flattening of EEG power rhythmicity has been associated with stress experiences in other rodent models [6, 10]. It remains difficult to ascribe with certainty biological significance to these individual changes spectral power, particularly when numerous changes occur in parallel. In the context of sleep, delta power is often viewed as a measure of sleep drive, with increased delta after extended wakefulness that decreases with sleep duration [60]. Similarly, theta power typically corresponds with wakefulness and increases with extended periods of wake, such as in sleep deprivation [62, 63]. Accordingly, decreased delta power in PACAP-treated mice during SWS might represent reduced wake-induced sleep drive, which aligns with findings in individuals diagnosed with PTSD, who often show decreased delta during NREM sleep [64]. Both delta and theta have also been implicated in attention and cognitive control [62]. Reduced gamma power during SWS in PACAP-treated mice might be related to subjective sleep duration, with reduced gamma during NREM sleep corresponding with overestimation of sleep duration in individuals with insomnia [63]. In CRF-treated mice, decreased delta and theta power during wakefulness might signify subtle homeostasis-related responses that are not reflected by major changes in sleep architecture. More work is needed in both humans and preclinical models to enable deeper insight into the meaning of these changes and how they might be used to diagnose psychiatric illness or predict impending mental health issues [12]. Together, our findings suggest that PACAP plays an important role in stress-induced changes of sleep architecture and associated biological rhythms, whereas CRF (at an anxiogenic-equivalent dose) contributes to depression-like changes in diurnal rhythms of body temperature and activity but is less involved in the regulation of vigilance states.

Analyses of baseline sleep prior to treatment revealed sex differences in duration of AW and SWS, but not REM sleep. Sex differences in sleep architecture have been previously reported in both humans and rodents [7, 63, 64]. Sleep alterations in females are largely dependent on sex-related hormones, estrous cycle in rodents and menstrual cycle in humans [65–67], which were not studied in these experiments. Variations in core body temperature can be utilized to determine estrous phase in female mice, but because the effect of estrous phase on sleep was not a primary goal of this work, the number of females in each phase at baseline was not explicitly controlled [56, 68]. Coincidental estrous phase alignment was insufficient to properly assess its role on sleep/wake durations, although sleep/wake durations of all females together were significantly different than those of males. Future studies could control estrous phase more explicitly, enabling more comprehensive conclusions.

Comparisons of sleep architecture after peptide infusions expressed as percentage of baseline did not reveal sex differences. This was unexpected, considering that sex differences in PACAP and CRF systems have been described, in addition to baseline sex differences in sleep found in this study. There are reports indicating sex differences in CRF receptor density in stress-sensitive brain areas (e.g., amygdala), as well as increased CRF activation after stress in females compared to males [37, 39, 41, 42]. Expression of the cognate PACAP receptor (PAC1R) is regulated by estrogen, and has been shown to vary across the female estrous cycle in rodents [20, 22]. In humans, PACAP is associated with depression in males and anxiety-related disorders in women [22, 23, 31]. Single nucleotide polymorphisms (SNPs) relating to PACAP and CRF receptors are associated with PTSD and symptom severity, particularly in women [22, 31, 69]. Furthermore, biological sex is a significant risk factor in stress- and fear-related psychiatric illnesses, with higher prevalence of PTSD, GAD, and MDD in women compared to men [35, 36, 70]. Despite these previous findings, we did not observe sex differences in sleep architecture as a result of PACAP or CRF under our testing conditions. To assess the overall effects of PACAP and CRF on sleep architecture with maximal statistical power, we combined males and females [58].

Our finding that PACAP treatment increases in both SWS and REM sleep aligns with prior reports indicating that ICV and intra-pons infusions of PACAP produce increases in REM sleep [46, 47]. Prior work also shows that CSDS increases REM sleep [10], and increased REM sleep duration in humans is a risk factor for depressive illness [60]. The sustained effects of PACAP on REM sleep at Day 7 also aligned with previous reports that PACAP effects are persistent. For example, the effects of systemic PACAP on acoustic startle persist for longer than a week, and PACAP infusions into the pons also cause long-lasting effects on sleep [21, 29, 30, 47]. We previously reported that changes in REM sleep as a result of CSDS also persist at least five days [10]—the longest time point tested—raising the possibility that PACAP plays a role in long-lasting changes to sleep architecture after this type of stress regimen. In addition, the paraventricular nucleus (PVN), a critical sleep region, and the parabrachial nucleus (PBn), an area associated with stress-sleep interactions, are both sites of PACAP production [27, 47, 56, 71]. PACAP is also involved in responses to light, acting along the neuronal pathway from the retina to the superchiasmatic nucleus (SCN) [72], representing another mechanism by which it may regulate sleep architecture. Together, these findings represent accumulating evidence that PACAP plays a key role in the acute and persistent effects of stress on sleep.

We did not observe strong effects of CRF treatment on sleep architecture, despite previous reports and evidence that CRF interacts with neural circuits involved in sleep [43–45, 50, 73]. While methodological variations could account for these differential findings, the dosage of CRF and time of day of CRF administration may be involved. In the current studies, dosages were selected on the basis of their ability to cause equivalent behavioral responses in the EPM, a widely-used and thoroughly-validated procedure for assessing anxiety-like behavior [57]. Region-specific infusions of CRF into the central amygdala of rats reportedly reduces REM sleep duration when provided at low but not high doses [50], suggesting an inverted U-shaped function. While our infusions were not region specific, it is possible that the dosage we used was too high, despite causing an anxiogenic-like response in the EPM equivalent to that seen with PACAP. In fear conditioning studies, ICV CRF enhances fear-related reductions in REM sleep in the dark phase of the light cycle, but did not alter light phase sleep immediately after treatment [43]. One of our primary goals was to examine the persistence of PACAP and CRF effects, considering differences in other behavioral endpoints. The effects of PACAP on sleep architecture were largely observed in the dark phase of the light cycle, as were the only observed CRF-induced changes in sleep. It is conceivable that the time point used for peptide infusion—early in the light cycle—could interfere with the dark phase effects of CRF. While we found that CRF produced a dark-phase change in SWS, it is possible that infusions later in the light phase of the light cycle, or during the dark phase, might elicit larger alterations than we observed in the present studies. In humans, intravenous CRF decreases SWS, increases wakefulness, and increases REM, particularly in women [74, 75]. The unsuccessful development of CRF antagonism monotherapy suggests that additional targets—such as PACAP systems—may be involved in the full scope of stress-sleep interactions [25, 76–80].

Consistent with the existing literature, our findings suggest that PACAP contributes to stress effects on sleep, by reducing time awake and increasing sleep duration. Direct comparison of PACAP and CRF provides clear evidence that PACAP administration produces acute alterations in sleep within 24 h, with persistent effects on REM sleep. While CRF shares some of these effects, its impact is not comparable to PACAP. Endpoints including sleep, body temperature, and locomotor activity have considerable translational value, as they are defined and measured in the same ways across species and are increasingly available in human studies through devices, such as smart phones and wearables [12]. Our findings raise the possibility that PACAP, but not CRF, plays a role in the persistence of stress-induced sleep dysregulation, which tend to be persistent and intractable in individuals with stress-related psychiatric conditions [8]. The ability to target and relieve stress-related sleep abnormalities may enable new and more effective approaches to treating psychiatric illness.

### Citation diversity statement

The authors have attested that they made efforts to be mindful of diversity in selecting the citations used in this article.
